# Optimizing Mechanical, Moisture‐Based, and Enzymatic Pretreatments to Improve Dehulling Outcomes and Nutritional Quality in Four US Dry Bean Market Classes

**DOI:** 10.1111/1750-3841.71342

**Published:** 2026-08-19

**Authors:** Winter Graham, Karen Cichy, Jeffrey Swada

**Affiliations:** ^1^ Department of Food Science and Human Nutrition Michigan State University East Lansing Michigan USA; ^2^ Sugarbeet and Bean Research Unit USDA‐ARS East Lansing Michigan USA

## Abstract

Dry beans (*Phaseolus vulgaris* L.) are a nutrient‐dense and sustainable protein source, with potential for use as a food ingredient, but strong adhesion between the seed coat and cotyledon limits processing efficiency. This study evaluated mechanical and moisture‐based pretreatments, with and without enzymatic supplementation, to improve dehulling performance across four major US market classes (black, pinto, navy, and Great Northern) and assessed the nutritional quality of resulting cotyledon and hull fractions. Small‐scale trials identified that moisture tempering to 40% combined with bran‐cracking was the primary driver of improved dehulling (dehulling index [DI] = 0.84 ± 0.01), with low enzyme addition providing a comparable DI (DI = 0.84 ± 0.01). Validation trials across four market classes confirmed that bran‐cracking with 40% moisture tempering achieved consistently high performance (DE = 82%–89%, DD = 95%–100%, DI = 0.64–0.68), while enzyme addition provided no statistically significant additional benefit at the validation scale. Compositional analysis showed that hulls contained 33.2 ± 1.5% crude fiber and 6%–16% protein, while cotyledons contained 12.4 ± 3.5% crude fiber and 19%–24% protein; across all pretreatments, cotyledon protein concentration remained stable, supporting its suitability as a consistent, protein‐dense ingredient stream. These findings demonstrate that moisture tempering combined with mechanical bran‐cracking constitutes a practical, enzyme‐free framework for enhancing dry bean dehulling while supporting value‐added utilization of hulls as functional, fiber‐rich ingredients.

**Practical Applications:**

This work identifies moisture tempering combined with mechanical bran‐cracking as the primary driver of improved dry bean dehulling performance—a practical, enzyme‐free pretreatment strategy applicable across multiple US market classes. By enabling cleaner separation and recovering fiber‐rich hull fractions as functional, clean‐label ingredients, this approach supports reduced waste and expanded ingredient opportunities in pulse‐processing operations.

**Abbreviations**
DD: degree of dehulling
DE: dehulling efficiency
DI: dehulling index
MC: moisture content (%)

## 1 Introduction

Dry beans (*Phaseolus vulgaris* L.) are globally important sources of plant protein, fiber, minerals, and bioactive compounds, contributing significantly to dietary quality and food security in both high‐ and low‐income regions (Singh et al. 2022; Mullins and Arjmandi 2021). Despite their nutritional value and sustainability advantages relative to other protein crops (Uebersax et al. 2023), dry beans remain underutilized as functional food ingredients compared to other pulses such as soy, chickpea, or pea (Magrini et al. 2023). This ingredient gap is driven largely by technological barriers in processing, particularly those associated with dehulling inefficiency and seed coat adhesion (Fernando 2017; Sreerama et al. 2009).

The bean seed coat is structurally complex, consisting of palisade, osteosclereid, and parenchyma layers that undergo compression and lignification during maturation (Hughes and Swanson 1985). These anatomical changes reduce cell separability and create a dense, impermeable barrier that strongly adheres to the cotyledon. The adhesion is mediated largely by pectin–hemicellulose cross‐linking in the middle lamella, which inhibits water uptake, decreases hydration rates, and impedes mechanical hull removal (Nguyen et al. 2026; Fernando 2017). As a result, most common bean market classes exhibit lower dehulling responsiveness than other pulses, contributing to mechanical losses, irregular split formation, and reduced processing yields (Sreerama et al. 2009).

To overcome these structural constraints, enzyme‐assisted pretreatments have shown promise in weakening hull–cotyledon adhesion. Carbohydrases such as cellulase, xylanase, and pectinase can hydrolyze key structural polysaccharides within the seed coat, improving permeability and reducing tissue integrity at the adhesion plane (Wood et al. 2022). Prior studies in pigeon pea and horse gram reported significant improvements in dehulling efficiency (DE) and cooking quality following enzymatic application (Dabhi et al. 2019; Sreerama et al. 2009). Enzymes also synergize with hydration, allowing water to penetrate structural tissues more effectively and accelerate softening kinetics (Offia and Madubuike 2015). Mechanical interventions, such as bran‐cracking, may further enhance enzyme efficacy by increasing surface area and creating microfractures for enzyme diffusion.

Beyond processing improvements, the valorization of bean hulls offers an emerging opportunity to reduce waste and develop new functional ingredients. Bean hulls, particularly those of pigmented varieties such as black beans, are rich in insoluble fiber, polyphenols, anthocyanins, and antioxidant compounds, often containing over 80%–90% of total seed phenolic content (Xu and Chang 2007). These bioactive‐rich hull fractions have demonstrated potential applications in dietary fiber enrichment, natural colorant development, and antioxidant supplementation. Despite this potential, hulls are often discarded in processing side streams, representing an opportunity for upcycling in future pulse dehulling processes.

To our knowledge, this study is the first to directly compare enzyme‐assisted and enzyme‐free mechanical pretreatments across four major US dry bean market classes within a single study while also assessing the compositional quality of both cotyledon and hull fractions. The objective of this study was to identify practical pretreatment strategies that improve dry bean dehulling performance and support value‐added utilization of the resulting fractions. Specifically, the study evaluated pretreatment variables in black beans to identify high‐performing conditions and then tested the transferability of selected treatments across black, pinto, navy, and Great Northern beans.

## 2 Materials and Methods

### 2.1 Materials

All beans across market classes were harvested from the 2024 summer crop for consistency. Dry black beans were obtained from ADM (Frankenmuth, MI, USA), pinto beans were obtained from Star of the West (Frankenmuth, MI, USA), and navy beans were obtained from Michigan State University's Saginaw Valley Research Farm and Extension Center (Frankenmuth, MI, USA). Great Northern beans were obtained from the University of Nebraska–Lincoln Department of Agronomy & Horticulture (Lincoln, NE, USA). Food‐grade cellulase and xylanase enzymes were obtained from Sunson Industry Group Co., Ltd (Beijing, China), and food‐grade pectinase was obtained from Purelife Biotech Co., Ltd (Shanghai, China). All other reagents were of analytical grade and obtained from Sigma–Aldrich (St. Louis, MO, USA) and included sodium dihydrogen phosphate (NaH_2_PO_4_) and disodium hydrogen phosphate (Na_2_HPO_4_).

### 2.2 Methods

#### 2.2.1 Small‐Scale Dehulling Trials (Black Beans)

Small‐scale pretreatment optimization was initially performed using black beans to refine dehulling pretreatments prior to validation trials. A total of 200 g of whole beans were allocated per treatment sample. All beans were visually inspected and cleaned to remove damaged seeds and debris before treatment application.

Treatments included (1) control (no pretreatment), (2) moisture adjustment only, (3) moisture adjustment with enzyme (low/medium/high), (4) moisture adjustment and bran‐cracking without enzyme, (5) moisture adjustment and bran‐cracking with enzyme (low/medium/high), and (6) enzyme soaking without bran‐cracking.

Following pretreatment, beans were dried under one of two conditions: (1) non‐roasting conditions: returned to within ±2% of starting moisture, or (2) roasting conditions: dried to <8% moisture content (MC).

**FIGURE 1:**
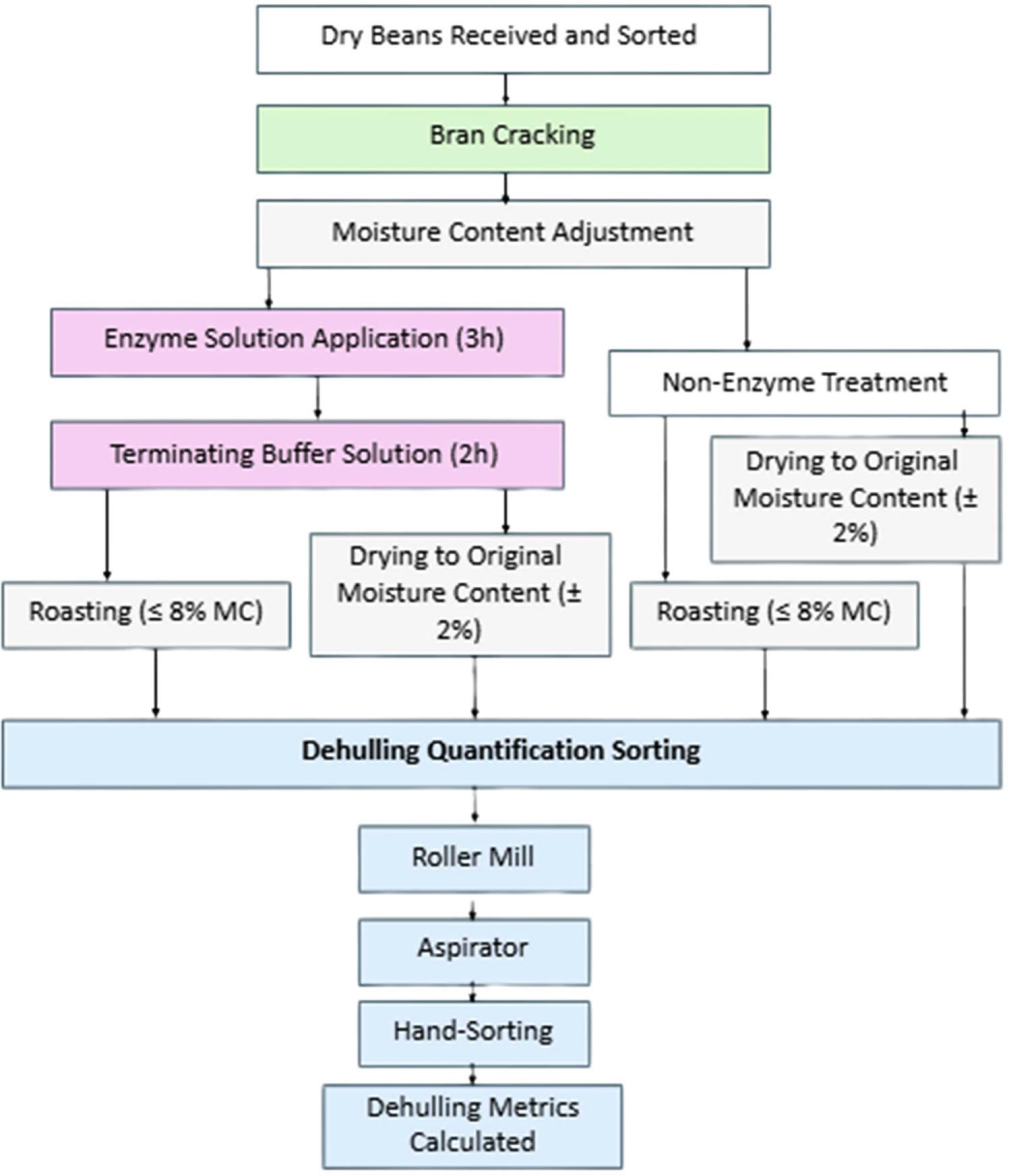
Flowchart outline of all dehulling treatments and dehulling quantification. Colored boxes indicate steps that were applied for specific treatment families: pink = enzyme treatment; green = bran‐cracking; gray = moisture content adjustment.

Dehulling was performed using a FERRODAY (Lincolnshire, IL, USA) roller mill (0.88 mm gap) and drying with a D‐20 Dehydrator from The Sausage Maker Inc. (Buffalo, NY, USA) at 160°F (71°C) for 5 h with moderate airflow until returning to within ±2% of the starting MC. Fractions were separated using a KICE Industries (Park City, KS, USA) 6DT4‐Rollaway Aspirator (0.5 W.C./in) in three passes and sieved (0.250 mm, #60). All fractions (dehulled cotyledons, non‐dehulled beans, hulls, fines) were weighed for dehulling metric calculations detailed in Section 2.2.7 and Figure 1.

#### 2.2.2 Validation Dehulling Trials (Black, Pinto, Navy, and Great Northern Beans)

To evaluate treatment transferability across market classes, selected pretreatments from small‐scale trials were applied to 400‐g validation batches of each bean type (black, pinto, navy, Great Northern). All treated samples were tempered to 40% moisture prior to pretreatment, as this condition provided the strongest performance improvements. Tempering procedures followed the methods of Fernando (2017), where water was incorporated into the sample using physical agitation, and are detailed in Section 2.2.4.

Moisture adjustment, bran‐cracking without enzyme, and bran‐cracking with low enzyme treatments followed the identical soaking, drying, and dehulling configurations used in small‐scale trials, with continuous roller‐mill throughput to accommodate larger volumes.

#### 2.2.3 Control—No Treatment

Untreated beans served as controls to establish baseline dehulling behavior. Control beans were milled directly using a FERRODAY roller mill (0.88 mm gap) with no moisture adjustment or enzyme exposure.

Hull separation was performed using a KICE Industries 6DT4 Rollaway Aspirator for three passes at 0.5 W.C./in, followed by manual sieving to collect fines.

#### 2.2.4 Moisture Adjustment Procedure

Initial MC was measured using an agraTronix MT‐16 Grain Moisture Tester (Streetsboro, OH, USA) in averaged, triplicate readings.

Beans were tempered with deionized water to reach the target MC following the methods of Fernando (2017), where water was incorporated into the sample using physical agitation, and MC was confirmed on a weight basis. After equilibration in sealed containers, beans were air‐dried at 160°F (71°C) for 5 h with moderate airflow until returning to within ±2% of the starting MC. Dried beans were then dehulled and fractionated as described above. The process can be seen outlined in Figure 1 and Table 1.

**TABLE 1:** Summary of treatment combinations and experimental stages used to evaluate dry bean dehulling performance.

Experimental stage	Bean market class(es)	Batch size	Treatment family	Moisture level	Bran‐cracking	Enzyme treatment	Drying condition
Control	Black; pinto; navy; great Northern	200 g	No pretreatment	Native moisture	No	No	N/A
Small‐scale screening	Black	200 g	Moisture adjustment only	30%, 40%, or 50% MC	No	No	Air‐dried to within ±2% of starting moisture
			Moisture adjustment + enzyme	30%, 40%, or 50% MC	No	Low, medium, or high enzyme concentration	Air‐dried to within ±2% of starting moisture or roasted to <8% MC
			Moisture adjustment + bran‐cracking	30%, 40%, or 50% MC	Yes	No	Air‐dried to within ±2% of starting moisture or roasted to <8% MC
			Moisture adjustment + bran‐cracking + enzyme	30%, 40%, or 50% MC	Yes	Low, medium, or high enzyme concentration	Air‐dried to within ±2% of starting moisture or roasted to <8% MC
Validation trials	Black; pinto; navy; Great Northern	400 g	Control	Native Moisture	No	No	N/A
			40% moisture + bran‐cracking	40% MC	Yes	No	Air‐dried to within ±2% of starting moisture
			40% moisture + bran‐cracking + low enzyme	40% MC	Yes	Low enzyme concentration	Air‐dried to within ±2% of starting moisture

*Note*: Low enzyme = 6 g xylanase, 3 g pectinase, and 3 g cellulase per 200 g bean sample. Medium enzyme = 11 g xylanase, 5.5 g pectinase, and 5.5 g cellulase per 200 g bean sample. High enzyme = 23 g xylanase, 11.5 g pectinase, and 11.5 g cellulase per 200 g bean sample.

Abbreviation: MC, moisture content.

#### 2.2.5 Bran‐Cracking (Non‐Enzyme) Treatment Procedure

For bran‐cracking, beans were coarsely fractured using a professional‐grade 14‐cup food processor from Cuisinart (Stamford, CT, USA) using 15‐s pulses to induce micro‐fractures in the seed coat. Cracked beans were tempered to target MC, air‐dried (160°F for 5 h), and milled. In a roasted variant, beans were instead dried at 250°F (121°C) for 5 h to achieve ≤8% MC (ideally ∼7%). Dried beans were then dehulled and fractionated as described above.

#### 2.2.6 Enzymatic Pretreatments

##### 2.2.6.1 Enzyme Concentrations

All enzymatic treatments used combinations of xylanase, cellulase, and pectinase. Concentrations were determined using preliminary testing conducted in the summer of 2024, where the dehulling index (DI) was calculated and assessed for the feasibility of research variables. Low‐concentration enzyme solutions contained 6 g of xylanase, 3 g of pectinase, and 3 g of cellulase. Medium concentration enzyme solutions contained 11 g of xylanase, 5.5 g of pectinase, and 5.5 g of cellulase. High‐concentration enzyme solutions contained 23 g of xylanase, 11.5 g of pectinase, and 11.5 g of cellulase. These specific concentrations were derived from preliminary dehulling‐index‐based feasibility testing rather than simple arithmetic scaling, as preliminary trials indicated that the selected ratios provided measurable differences in dehulling performance across concentration levels. Enzyme activities, where available from manufacturer specifications, are noted parenthetically: xylanase (approximately 50,000 U/g; 1500 U/g bean at low concentration), pectinase (approximately 3000 U/g; 45 U/g bean at low concentration), and cellulase (approximately 700 U/g; 10.5 U/g bean at low concentration); concentrations are reported per 200 g sample batch.

##### 2.2.6.2 Enzyme Soaking Procedure

Beans were cleaned, sorted, and subjected to bran‐cracking and tempering prior to enzyme application. Each 200‐g sample was soaked in 250 mL of enzyme solution (low/medium/high concentration) for 3 h at room temperature, with manual agitation every 30 min.

Reactions were stopped by soaking beans in 250 mL of 0.1 M sodium phosphate buffer (pH 7.0) for 2 h, following slightly modified methods of Sreerama et al. (2009). This composite formulation and reaction time were held constant across all bean market classes and are detailed in Figure 1 and Table 1. Beans were then either air‐dried to within ±2% of original MC following the procedure described in Section 2.2.4 (160°F [71°C] for 5 h with moderate airflow) or roasted at 250°F (121°C) to achieve <8% MC.

All treatments across small‐scale and validation‐scale trials are summarized in Table 1.

#### 2.2.7 Dehulling Quantification Sorting and Metrics

##### 2.2.7.1 Dehulling Quantification Sorting Procedure

**FIGURE 2:**
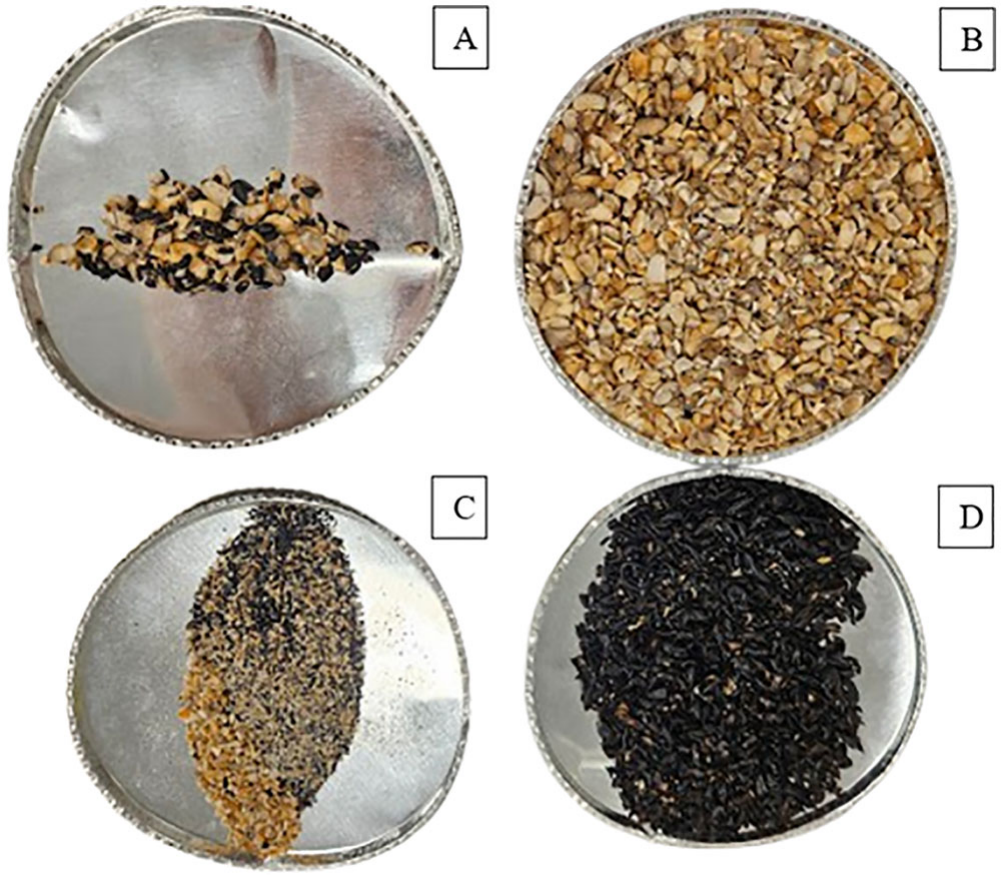
Final products obtained from successful dehulling pre‐treatments and quantification sorting. (A) Non‐dehulled beans. (B) Dehulled cotyledon. (C) Fine powder/brokens. (D) Hulls.

Following each pretreatment, samples were processed through dehulling and separation stages as summarized in Figure 1. Beans were dehulled using a roller mill (0.88 mm gap), followed by three aspirator passes at 0.5 W.C./in to remove hull fractions. Residual materials were hand‐sorted to separate successful dehulled beans (clean cotyledon), non‐dehulled beans, hulls, and fines/broken fractions using a size 40‐mesh sieve, which can be seen in Figure 2.

Fraction masses were recorded and used to calculate DE, degree of dehulling (DD), and DI as defined in Section 2.2.7.2.

##### 2.2.7.2 Dehulling Metrics Calculation Procedure

Quantitative evaluation of dehulling performance was conducted following the combined framework of Sreerama et al. (2009), Fernando (2017), and Dabhi et al. (2019), with modifications for the roller‐mill and KICE aspirator system used in this study. After each dehulling run, all fractions—dehulled cotyledons (*W*_2_), non‐dehulled beans (*W*_3_), hulls (*W*_h_), and fines/broken powder (*W*_b_)—were sorted, collected, and weighed to the nearest 0.01 g. See Figure 2 for a visual example of each fraction within each dehulling sample. The initial sample weight (*W*_1_) represented the total mass of beans processed for that replicate. Each metric was calculated as follows.

###### 2.2.7.2.1 DE (%)

DE estimates the yield of usable cotyledons relative to the initial sample mass and was calculated as

(1) $$\mathrm{DE}\%=\frac{W_{2}}{W_{1}}\;\times 100,$$

where *W*_2_ is the mass of dehulled cotyledons and *W*_1_ is the initial sample mass. Higher DE values correspond to more efficient recovery of intact cotyledons with minimal material loss.

###### 2.2.7.2.2 DD (%)

The proportion of dehulled cotyledon relative to the original bean mass was calculated as:

(2) $$\mathrm{DD}\%=\frac{\left(W_{1-}W_{3}\right)}{W_{1}}\times 100,$$

where a higher DD value indicates a greater percentage of hull removal.

###### 2.2.7.2.3 DI

The DI provides an integrated measure of dehulling completeness and product quality:

(3) $$\mathrm{DI}=\frac{\left(\left(W_{2-}W_{h}\right)-\left(W_{3-}W_{b}\right)\right)}{W_{1}},$$

where values theoretically range from −1 to +1, with +1 representing complete dehulling (all cotyledons and hulls separated, no fines or non‐dehulled beans) and −1 indicating poor dehulling (predominantly intact or fragmented beans).

All calculations were performed in triplicate for each treatment group and averaged to obtain mean ± standard deviation. Dehulling metrics were used to compare the effects of pretreatment type, moisture level, and enzyme application across bean classes.

Although DE, DD, and DI were calculated separately, the three metrics describe related aspects of the same dehulling response. DE primarily reflects recovery of usable cotyledon material relative to the original sample mass, DD reflects the extent of hull removal, and DI integrates separation completeness with losses associated with fines, broken material, and non‐dehulled beans. Therefore, DE and DD provide interpretable component measures, while DI serves as the primary overall indicator of dehulling performance. In Section 3, these metrics are interpreted together rather than as independent outcomes to avoid redundant discussion and to emphasize treatment‐level processing effects.

#### 2.2.8 Proximate Composition and Nutritional Analyses

##### 2.2.8.1 Crude Protein (LECO Combustion Method)

Cotyledon and hull fractions collected after dehulling were dried to constant weight, finely ground using a Kinematica hammer mill (PX—MFC 90D) (Kinematica, Bohemia, NY, USA) equipped with a 0.5‐mm screen, and analyzed for nitrogen content using a LECO FP‐828 Nitrogen/Protein Determinator (LECO Corporation, St. Joseph, MI, USA). Approximately 0.15 g of sample was combusted under pure oxygen, using AACC Method 46‐30 (AACC International 2015a), and % nitrogen was quantified via thermal conductivity detection. Crude protein was calculated using the Jones factor of 6.25. All samples were analyzed in triplicate, with calibration standards and blanks included in each analytical batch.

##### 2.2.8.2 Crude Fiber Determination

Crude fiber content was determined according to AACC Method 32‐10.01 (AACC International 2015b) by the University of Missouri Agricultural Experiment Station Chemical Laboratories (ESCL). Method 32‐10.01 is a gravimetric method based on sequential acid and alkali digestion following the classical Weende approach. Approximately 1 g of defatted, ground sample (<1 mm particle size) was boiled in 1.25% H_2_SO_4_ for 30 min, filtered, washed, and subsequently boiled in 1.25% NaOH for 30 min. The residue was filtered, washed with hot water and acetone, dried at 105°C to constant weight, weighed, and then ignited in a muffle furnace at 550°C for ≥3 h. Crude fiber (%) was calculated as the percentage weight loss upon ashing of the dried residue. This method measures the indigestible cell wall components, cellulose, lignin, and hemicellulosic residues that remain after digestion.

#### 2.2.9 Statistical Analysis

##### 2.2.9.1 Dehulling Metrics Statistical Analysis

All calculations were performed in triplicate for each treatment group and averaged to obtain mean ± standard deviation (*n* = 3). Dehulling metrics were used to compare the effects of pretreatment type, moisture level, and enzyme application across bean classes.

All statistical analyses were performed in RStudio using R version 4.4.2 (Posit, Boston, MA, USA). Dehulling responses (DE, DD, and DI) were summarized as mean ± SD. A significance level of *α* = 0.05 was used for all inferential tests. For single‐factor comparisons (e.g., treatment family within a bean market class), one‐way ANOVA with Tukey's HSD post hoc testing was performed. For multifactor experiments (e.g., Treatment family × Moisture level × Bran‐cracking), three‐way ANOVA with Tukey's HSD post hoc testing was performed. Figures present mean ± standard error (SE), and distinct letters above bars denote statistically significant differences among treatments (Tukey HSD, *p* < 0.05).

##### 2.2.9.2 Proximate Composition and Nutritional Statistical Analysis

All protein and fiber data were analyzed in RStudio (R version 4.4.2) and expressed as mean ± standard deviation (*n* = 3). A one‐way analysis of variance (ANOVA) was used to evaluate the effect of the three pretreatments (no treatment = Control, 40% moisture with bran‐cracking = No enzyme, and 40% moisture with bran‐cracking and enzyme = Enzyme) on each nutritional parameter within bean samples where applicable.

When significant differences were detected (*p* < 0.05), Tukey's honest significant difference (HSD) post hoc test was performed to identify pairwise differences among treatment means. Figures present mean ± standard error (SE), and distinct letters above bars denote statistically significant differences among treatments (Tukey HSD, *p* < 0.05).

## 3 Results and Discussion

### 3.1 Effects of Pretreatments on Small‐Scale Dehulling Performance

Small‐scale black bean trials showed that moisture tempering and bran‐cracking were the dominant contributors to improved dehulling performance, while enzyme addition provided a smaller incremental benefit under selected conditions. Across DE, DD, and DI, treatments incorporating 40% moisture and bran‐cracking consistently outperformed untreated controls, indicating that hydration combined with mechanical disruption substantially improved hull release and cotyledon recovery as seen in Table 2.

**TABLE 2:** Small‐scale black bean dehulling performance by treatment combination.

Treatment description	Moisture level	Enzyme level	Bran‐cracking	Drying condition	DE (%) mean ± SE	DD (%) mean ± SE	DI mean ± SE
Control (no pretreatment)	Native	None	No	No drying/roasting	48.3 ± 0.5	63.7 ± 0.8	0.13 ± 0.02
Moisture adjustment only	30%	None	No	Air‐dried to starting MC	66.2 ± 2.9^b^	80.0 ± 3.6^b^	0.47 ± 0.06^b^
Moisture adjustment only	40%	None	No	Air‐dried to starting MC	70.1 ± 1.7^b^	84.8 ± 0.6^b^	0.54 ± 0.03^b^
Moisture adjustment only	50%	None	No	Air‐dried to starting MC	79.0 ± 0.4^a^	94.3 ± 0.6^a^	0.75 ± 0.01^a^
Moisture adjustment + bran‐cracking	30%	None	Yes	Air‐dried to starting MC	80.0 ± 0.4^a^	95.0 ± 0.4^a^	0.77 ± 0.01^b^
Moisture adjustment + bran‐cracking	40%	None	Yes	Air‐dried to starting MC	82.2 ± 0.1^a^	97.0 ± 0.4^a^	0.84 ± 0.01^a^
Moisture adjustment + bran‐cracking	50%	None	Yes	Air‐dried to starting MC	82.1 ± 1.0^a^	97.2 ± 0.8^a^	0.81 ± 0.01^a^
Moisture adjustment + bran‐cracking + roasting	30%	None	Yes	Roasted to <8% MC	81.8 ± 0.8^a^	96.7 ± 0.4^a^	0.81 ± 0.02^a^
Moisture adjustment + bran‐cracking + roasting	40%	None	Yes	Roasted to <8% MC	79.4 ± 0.8^a^	93.7 ± 0.9^b^	0.78 ± 0.02^a^
Moisture adjustment + bran‐cracking + roasting	50%	None	Yes	Roasted to <8% MC	81.7 ± 0.4^a^	97.3 ± 0.6^a^	0.79 ± 0.01^a^
Low enzyme + moisture adjustment	30%	Low	No	Air‐dried to starting MC	80.2 ± 0.5^a^	95.8 ± 0.7^a^	0.79 ± 0.01^a^
Low enzyme + moisture adjustment	40%	Low	No	Air‐dried to starting MC	80.2 ± 0.3^a^	97.1 ± 0.2^a^	0.80 ± 0.01^a^
Low enzyme + moisture adjustment	50%	Low	No	Air‐dried to starting MC	80.6 ± 0.5^a^	96.4 ± 0.5^a^	0.79 ± 0.01^a^
Low enzyme + moisture adjustment + bran‐cracking	30%	Low	Yes	Air‐dried to starting MC	75.1 ± 1.6^b^	91.0 ± 1.8^b^	0.72 ± 0.03^b^
Low enzyme + moisture adjustment + bran‐cracking	40%	Low	Yes	Air‐dried to starting MC	83.5 ± 0.3^a^	97.9 ± 0.5^a^	0.84 ± 0.01^a^
Low enzyme + moisture adjustment + bran‐cracking	50%	Low	Yes	Air‐dried to starting MC	82.8 ± 0.9^a^	97.9 ± 0.3^a^	0.82 ± 0.01^a^
Low enzyme + moisture adjustment + bran‐cracking + roasting	30%	Low	Yes	Roasted to <8% MC	79.2 ± 1.6^a^	92.9 ± 2.3^a^	0.75 ± 0.04^a^
Low enzyme + moisture adjustment + bran‐cracking + roasting	40%	Low	Yes	Roasted to <8% MC	81.3 ± 1.0^a^	95.9 ± 1.7^a^	0.81 ± 0.03^a^
Low enzyme + moisture adjustment + bran‐cracking + roasting	50%	Low	Yes	Roasted to <8% MC	80.5 ± 0.4^a^	95.2 ± 0.9^a^	0.80 ± 0.02^a^
Medium enzyme + moisture adjustment	30%	Medium	No	Air‐dried to starting MC	79.2 ± 0.7^a^	95.6 ± 0.5^ab^	0.77 ± 0.01^a^
Medium enzyme + moisture adjustment	40%	Medium	No	Air‐dried to starting MC	78.8 ± 0.9^a^	94.7 ± 0.5^b^	0.75 ± 0.01^a^
Medium enzyme + moisture adjustment	50%	Medium	No	Air‐dried to starting MC	80.5 ± 0.5^a^	96.8 ± 0.3^a^	0.79 ± 0.01^a^
Medium enzyme + moisture adjustment + bran‐cracking	30%	Medium	Yes	Air‐dried to starting MC	78.0 ± 0.6^a^	95.3 ± 1.4^a^	0.79 ± 0.02^a^
Medium enzyme + moisture adjustment + bran‐cracking	40%	Medium	Yes	Air‐dried to starting MC	78.7 ± 1.7^a^	96.0 ± 1.7^a^	0.80 ± 0.03^a^
Medium enzyme + moisture adjustment + bran‐cracking	50%	Medium	Yes	Air‐dried to starting MC	76.5 ± 2.1^a^	91.5 ± 2.3^a^	0.70 ± 0.05^a^
Medium enzyme + moisture adjustment + bran‐cracking + roasting	30%	Medium	Yes	Roasted to <8% MC	69.6 ± 3.6^ab^	85.3 ± 3.6^ab^	0.58 ± 0.08^ab^
Medium enzyme + moisture adjustment + bran‐cracking + roasting	40%	Medium	Yes	Roasted to <8% MC	66.3 ± 0.7^b^	80.2 ± 0.8^b^	0.50 ± 0.02^b^
Medium enzyme + moisture adjustment + bran‐cracking + roasting	50%	Medium	Yes	Roasted to <8% MC	78.3 ± 0.4^a^	93.3 ± 0.6^a^	0.76 ± 0.00^a^
High enzyme + moisture adjustment	30%	High	No	Air‐dried to starting MC	80.2 ± 0.5^a^	95.2 ± 0.2^a^	0.76 ± 0.01^a^
High enzyme + moisture adjustment	40%	High	No	Air‐dried to starting MC	80.1 ± 0.4^a^	95.9 ± 0.5^a^	0.77 ± 0.01^a^
High enzyme + moisture adjustment	50%	High	No	Air‐dried to starting MC	78.5 ± 0.9^a^	95.7 ± 0.5^a^	0.77 ± 0.01^a^
High enzyme + moisture adjustment + bran‐cracking	30%	High	Yes	Air‐dried to starting MC	74.5 ± 2.1^b^	91.1 ± 2.9^a^	0.70 ± 0.06^a^
High enzyme + moisture adjustment + bran‐cracking	40%	High	Yes	Air‐dried to starting MC	77.8 ± 1.6^ab^	94.6 ± 2.8^a^	0.78 ± 0.04^a^
High enzyme + moisture adjustment + bran‐cracking	50%	High	Yes	Air‐dried to starting MC	81.5 ± 0.4^a^	96.5 ± 0.9^a^	0.82 ± 0.01^a^
High enzyme + moisture adjustment + bran‐cracking + roasting	30%	High	Yes	Roasted to <8% MC	75.5 ± 1.8^a^	90.8 ± 2.3^a^	0.70 ± 0.04^a^
High enzyme + moisture adjustment + bran‐cracking + roasting	40%	High	Yes	Roasted to <8% MC	73.5 ± 4.6^a^	88.1 ± 4.7^a^	0.64 ± 0.10^a^
High enzyme + moisture adjustment + bran‐cracking + roasting	50%	High	Yes	Roasted to <8% MC	75.1 ± 2.7^a^	91.0 ± 3.6^a^	0.70 ± 0.08^a^

*Note*: Values are presented as mean ± standard error (*n* = 3). Superscript letters compare moisture levels within the same treatment family and metric using Tukey HSD (*p* < 0.05); means sharing a letter are not significantly different. Control values are shown for reference and were not included in moisture‐level comparisons.

The strongest small‐scale response was observed for the 40% moisture bran‐crack + low‐enzyme treatment, which produced DE = 83.5 ± 0.3%, DD = 97.9 ± 0.5%, and DI = 0.84 ± 0.01. The enzyme‐free bran‐crack treatment performed comparably, achieving DE = 82.2 ± 0.1%, DD = 97.0 ± 0.4%, and DI = 0.84 ± 0.01. In comparison, the untreated control remained lower across all three metrics, with DE = 48.3 ± 0.5%, DD = 63.7 ± 0.8%, and DI = 0.13 ± 0.02. These results indicate that the major improvement was attributable to moisture‐assisted mechanical weakening of the seed coat, with low‐enzyme addition producing a comparable DI to the enzyme‐free treatment at the 40% moisture level. The increase in overall dehulling efficacy seen with the introduction of bran‐cracking is detailed in Table 4.

The similar trends observed across DE, DD, and DI suggest that these indices captured overlapping treatment effects rather than independent responses. DE reflected improved cotyledon recovery, DD reflected more complete hull removal, and DI summarized the overall quality and uniformity of separation. Because all three metrics ranked the 40% moisture bran‐crack treatments among the highest‐performing conditions, DI was used as the primary integrated indicator of treatment performance, while DE and DD were interpreted as supporting measures.

Roasted treatments generally performed less favorably than non‐roasted treatments. This pattern may reflect reduced hydration‐associated softening or changes in seed coat texture during drying, although direct structural measurements were not collected to confirm the underlying mechanism. Therefore, the lower performance of roasted samples should be interpreted as a processing response rather than evidence of a specific structural change.

Overall, the small‐scale trials identified 40% moisture tempering combined with bran‐cracking as the most practical and reproducible pretreatment framework. Low‐enzyme addition produced the highest numerical DI at this scale, but the comparatively strong performance of the enzyme‐free bran‐crack treatment suggested that mechanical disruption and moisture conditioning were the primary drivers of dehulling improvement.

### 3.2 Validation Trials Across Bean Market Classes

**FIGURE 3:**
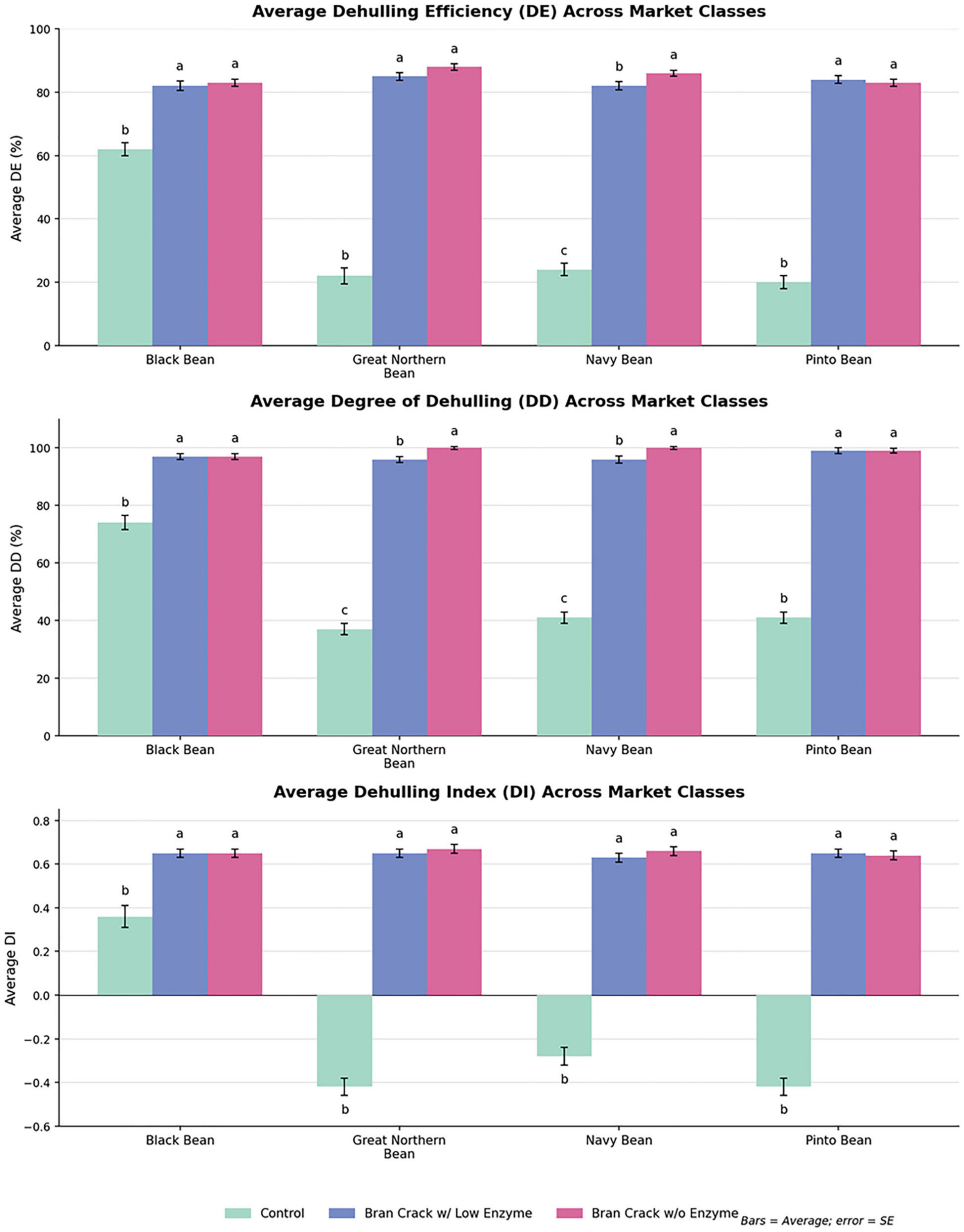
Validation‐scale dehulling efficiency (DE), degree of dehulling (DD), and dehulling index (DI) across market classes. Error bars = SE. Different letters on data bars represent significant differences between processing methods (*n* = 3) for average DE%, DD%, or DI (Tukey's HSD, *p* < 0.05).

Validation trials across black, pinto, navy, and Great Northern beans confirmed that the optimized moisture tempering and bran‐cracking strategy improved dehulling performance across market classes. As shown in Figure 3, both bran‐crack treatments, with and without enzyme addition, produced substantially higher DE, DD, and DI values than untreated controls. However, enzyme addition did not provide a consistent statistically significant advantage over the enzyme‐free bran‐crack treatment at the validation scale, indicating that moisture‐assisted mechanical disruption was the primary driver of improved dehulling.

Across market classes, treated samples achieved high dehulling performance, with DE values generally in the 82%–89% range, DD values in the 95%–100% range, and DI values in the 0.64–0.68 range. In contrast, untreated controls showed substantially poorer separation, with lower cotyledon recovery and less complete hull removal. These results demonstrate that controlled hydration and bran‐cracking improved both the amount of recoverable cotyledon material and the uniformity of hull separation.

The relationship among DE, DD, and DI was consistent with the small‐scale results. DE and DD provided useful component measures of cotyledon recovery and hull removal, while DI captured the combined effect of separation completeness and material losses. Because the three indices followed similar treatment trends, discussing them together provides a clearer interpretation than treating them as independent outcomes. The validation results, therefore, support the use of DI as the primary integrated performance measure, with DE and DD retained as supporting indicators.

The absence of a consistent enzyme benefit at the validation scale is important for practical interpretation. Enzymatic pretreatment required soaking, incubation, reaction termination, and drying, which add processing time, water use, reagent handling, and cost. Because the enzyme‐free bran‐crack treatment produced comparable performance across market classes, the enzyme‐free approach appears more practical for future scale‐up than the enzyme‐assisted pathway. Enzymes may still be useful under specific conditions, such as reduced mechanical shear or bean classes with unusually strong hull adhesion, but the present results do not support enzyme addition as necessary for high dehulling performance under the tested validation conditions.

Differences among market classes likely reflect variation in seed size, hull structure, and hull–cotyledon adhesion strength. However, because direct structural measurements were not collected, these explanations should be interpreted as hypotheses rather than confirmed mechanisms. Overall, the validation trials demonstrate that 40% moisture tempering combined with bran‐cracking provides a reproducible, enzyme‐free pretreatment strategy for improving dry bean dehulling across multiple US market classes.

### 3.3 Nutritional and Compositional Quality of Cotyledon and Hull Fractions

#### 3.3.1 Protein Composition of Cotyledon and Hull Fractions

Protein concentrations of cotyledon and hull fractions for all four market classes are summarized in Table 3. Across all market classes and treatments, cotyledons contained substantially higher protein concentrations than hull fractions, confirming that dehulling produced a protein‐rich cotyledon stream and a compositionally distinct hull stream. Pinto cotyledons generally contained the highest protein concentrations, while black and Great Northern cotyledons were lower but remained within the expected protein range for dry bean fractions (Tukey HSD, *p* < 0.05).

**TABLE 3:** Protein and crude fiber composition of cotyledon and hull fractions across dry bean market classes and pretreatments.

Market class	Treatment	Cotyledon protein (% DM)	Hull protein (% DM)	Cotyledon crude fiber (% W/W)	Hull crude fiber (% W/W%)
Black	Control	19.69 ± 0.27^b^	15.19 ± 0.33^a^	8.76 ± 6.00^a^	18.90 ± 4.76^a^
Black	40% MC + bran‐cracking	21.69 ± 0.24^a^	10.88 ± 0.28^b^	7.68 ± 2.94^a^	23.40 ± 3.51^a^
Black	40% MC + bran‐cracking + low enzyme	19.56 ± 0.31^b^	6.12 ± 0.36^c^	15.01 ± 3.24^a^	26.90 ± 0.70^a^
Navy	Control	21.00 ± 0.25^a^	13.06 ± 0.29^a^	10.83 ± 2.63^a^	29.62 ± 3.05^a^
Navy	40% MC + bran‐cracking	20.89 ± 0.32^a^	10.38 ± 0.34^b^	11.29 ± 2.94^a^	29.49 ± 1.11^a^
Navy	40% MC + bran‐cracking + low enzyme	19.31 ± 0.33^b^	10.50 ± 0.37^b^	16.32 ± 10.11^a^	24.12 ± 0.25^b^
Pinto	Control	24.25 ± 0.29^a^	11.31 ± 0.30^a^	9.44 ± 1.10^a^	19.98 ± 9.87^a^
Pinto	40% MC + bran‐cracking	23.44 ± 0.32^b^	9.69 ± 0.35^b^	9.01 ± 4.60^a^	29.05 ± 2.48^a^
Pinto	40% MC + bran‐cracking + low enzyme	20.50 ± 0.26^c^	8.81 ± 0.33^c^	8.29 ± 6.48^a^	27.82 ± 1.69^a^
Great Northern	Control	20.12 ± 0.28^ab^	16.50 ± 0.32^a^	13.18 ± 3.37^a^	29.90 ± 4.24^a^
Great Northern	40% MC + bran‐cracking	20.69 ± 0.30^a^	10.00 ± 0.27^b^	14.57 ± 3.81^a^	27.11 ± 2.27^a^
Great Northern	40% MC + bran‐cracking + low enzyme	19.69 ± 0.34^b^	9.94 ± 0.36^b^	11.68 ± 4.26^a^	24.45 ± 2.14^a^

*Note*: Values are presented as mean ± standard deviation (*n* = 3). Protein values are reported as % dry matter (%DM), and fiber values are reported as mean crude fiber content (% W/W). Letters compare treatments within the same market class and fraction using one‐way ANOVA with Tukey HSD (*p* < 0.05); means sharing a letter are not significantly different.

**TABLE 4:** Small‐scale black bean's bran‐cracking (BC) versus treatment family averaged across moisture level families.

Enzyme family	Moisture	DI (dehulling index)			DD% (degree of dehulling)			DE% (dehulling efficiency)			
		BC alone (NE‐NR)	Enzyme + BC	Δ	BC alone (NE‐NR)	Enzyme + BC	Δ	BC alone (NE‐NR)	Enzyme + BC	Δ
HE‐NR	LM	0.767 ± 0.052	0.697 ± 0.249	+0.070	94.96 ± 1.80	91.12 ± 12.33	+3.84	79.96 ± 1.82	74.53 ± 9.01	+5.44
	MM	0.870 ± 0.044	0.783 ± 0.180	+0.087	97.54 ± 0.79	94.59 ± 11.95	+2.95	83.09 ± 1.03	77.76 ± 6.89	+5.34
	HM	0.807 ± 0.038	0.820 ± 0.066	−0.013	97.24 ± 3.51	96.52 ± 3.76	+0.72	82.09 ± 4.17	81.53 ± 1.65	+0.56
LE‐NR	LM	0.767 ± 0.052	0.720 ± 0.131	+0.047	94.96 ± 1.80	91.03 ± 7.58	+3.93	79.96 ± 1.82	75.13 ± 7.08	+4.84
	MM	0.870 ± 0.044	0.850 ± 0.013	+0.020	97.54 ± 0.79	98.04 ± 0.60	−0.49	83.09 ± 1.03	84.33 ± 1.07	−1.24
	HM	0.807 ± 0.038	0.820 ± 0.127	−0.013	97.24 ± 3.51	97.69 ± 5.53	−0.44	82.09 ± 4.17	82.34 ± 16.65	−0.25
ME‐NR	LM	0.767 ± 0.052	0.790 ± 0.086	−0.023	94.96 ± 1.80	95.30 ± 5.93	−0.34	79.96 ± 1.82	78.03 ± 2.63	+1.94
	MM	0.870 ± 0.044	0.797 ± 0.123	+0.073	97.54 ± 0.79	96.02 ± 7.14	+1.53	83.09 ± 1.03	78.72 ± 7.47	+4.37
	HM	0.807 ± 0.038	0.700 ± 0.221	+0.107	97.24 ± 3.51	91.45 ± 9.71	+5.79	82.09 ± 4.17	76.45 ± 9.14	+5.63

*Note*: Values indicate means ± 95% confidence intervals. All comparisons restricted to non‐roasted (NR) treatments. LM = low moisture (30%); MM = medium moisture (40%); HM = high moisture (50%). Δ = BC alone minus Enzyme + BC; positive values indicate that bran‐cracking alone outperforms the enzyme with bran‐cracking treatment for that moisture level. BC alone = Bran‐cracking with no enzyme and no roasting (NE‐NR). HE = high enzyme; LE = low enzyme, and ME = medium enzyme, all without roasting (NR).

Across pretreatments, the No Enzyme condition (40% moisture + bran‐cracking) retained cotyledon protein concentrations similar to or slightly higher than the enzyme‐treated condition, indicating that enzyme addition did not improve cotyledon protein retention. This pattern supports the broader interpretation that moisture‐assisted mechanical pretreatment improved separation performance without compromising cotyledon protein quality.

Enzyme‐treated samples showed lower hull protein values in some market classes, including black and navy beans. This may reflect reduced cotyledon carryover into the hull fraction or processing‐related redistribution during soaking and drying; however, direct structural or compositional evidence would be needed to confirm the mechanism. Therefore, these changes should be interpreted cautiously as treatment‐associated compositional differences rather than direct evidence of enzymatic removal of residual cotyledon material.

Overall, cotyledon protein concentration remained high and relatively stable across all pretreatments (19%–24% protein), supporting the suitability of the cotyledon fraction as a consistent protein‐dense ingredient stream. The corresponding hull fractions retained lower protein concentrations and therefore represent a distinct co‐product stream better positioned for fiber‐focused applications. These outcomes are consistent with prior work showing that dehulling concentrates protein in the cotyledon fraction while processing primarily influences hull‐associated fiber and phenolic components (Choi et al. 2023; Viana and English 2022; Acquah et al. 2021).

#### 3.3.2 Crude Fiber Content of Cotyledon and Hull Fractions

Average crude fiber contents (% W/W) of cotyledon and hull fractions are illustrated in Table 3. As anticipated, hulls exhibited substantially greater fiber content than cotyledons across all market classes and treatments. The paired‐comparison plots demonstrate this pattern consistently, with hull values typically two to four times higher than their respective cotyledons.

Treatment effects on crude fiber were limited compared with the clear fraction‐level differences between cotyledons and hulls. Cotyledon fiber content did not differ significantly among treatments within any market class, and hull fiber content was generally stable across treatments, with the exception of navy bean hulls, where enzyme‐treated samples were lower than the control and no‐enzyme groups (Tukey HSD, *p* < 0.05). Because several cotyledon and hull fiber measurements showed high within‐group variability, these treatment‐level differences should be interpreted cautiously.

The more important compositional implication is that dehulling consistently separated a protein‐rich cotyledon fraction from a fiber‐rich hull fraction. This aligns with prior reports that dehulling reduces fiber in edible cotyledon fractions while increasing the relative concentration of protein‐rich material (Choi et al. 2023; Acquah et al. 2021). From an ingredient development perspective, the cotyledon fraction may provide a more protein‐dense base for pulse ingredients, while the hull fraction may be better suited for fiber enrichment or other value‐added applications.

The enzyme‐associated reduction in navy bean hull fiber may reflect redistribution of insoluble wall materials during soaking, drying, and separation rather than direct enzymatic degradation. Because equivalent trends were not observed consistently across market classes, additional replication and direct structural measurements would be needed to determine whether treatment‐specific fiber redistribution is biologically meaningful.

Bean seed coats and cotyledons also contain antinutritional factors, including phenolic compounds, phytates, and enzyme inhibitors, that may influence hydration behavior, enzyme accessibility, and cell‐wall interactions during pretreatment. Similar antinutritional factor changes have been reported in other processed and dehulled legume systems (Olaleye et al. 2020). These compounds were not quantified in the present study, so their contribution to dehulling performance cannot be directly determined. Future work should evaluate whether market class differences in antinutritional composition or enzyme inhibitory activity partly explain variation in responsiveness to enzyme‐assisted and enzyme‐free dehulling treatments.

## 4 Conclusion

This study demonstrates that moisture tempering to 40% combined with mechanical bran‐cracking was the primary driver of improved dry bean dehulling performance across major US market classes. In small‐scale trials, low‐enzyme supplementation and the enzyme‐free bran‐crack treatment produced comparable performance at 40% moisture, indicating that hydration and mechanical disruption were the main contributors to hull separation.

Validation trials confirmed that bran‐cracking with 40% moisture tempering improved DE, DD, and DI across black, pinto, navy, and Great Northern beans. Enzyme addition did not provide a consistent advantage over the enzyme‐free treatment at the validation scale, supporting the enzyme‐free mechanical pathway as the more reproducible and practically viable approach under the tested conditions.

Compositional analyses further showed that optimized dehulling did not compromise cotyledon quality. Cotyledon fractions retained high protein concentrations, while hull fractions remained fiber rich, supporting the potential use of both streams as value‐added ingredients.

From an applied standpoint, the enzyme‐free bran‐cracking‐plus‐tempering protocol is the most practical candidate for future scale‐up because it avoids the soaking, incubation, stopping reagent, and drying requirements associated with enzyme‐assisted pretreatment. Future work should evaluate the economics of this enzyme‐free protocol at larger batch processing conditions and determine whether bean chemical composition, including antinutritional factors such as tannins and phytic acid, contributes to market class differences in enzyme responsiveness.

## Author Contributions

**Winter Graham**: conceptualization, investigation, formal analysis, visualization, writing – original draft, data curation. **Karen Cichy**: conceptualization, funding acquisition, supervision, writing – review and editing. **Jeffrey Swada**: conceptualization, funding acquisition, supervision, writing – review and editing.

## Conflicts of Interest

The authors declare no conflicts of interest.

## Data Availability

Data are available upon request from the corresponding author.

## References

[jfds71342-bib-0001] AACC International . 2015a. “Method 46‐30.01. Crude Protein‐Combustion Method.” In Approved Methods of Analysis. 11th ed. AACC International.

[jfds71342-bib-0002] AACC International . 2015b. “Method 32‐10.01. Crude Fiber in Flours, Feeds and Feedstuffs.” Approved Methods of Analysis. 11th ed. AACC International.

[jfds71342-bib-0003] Acquah, C. , G. Ohemeng‐Boahen , K. A. Power , and S. M. Tosh . 2021. “The Effect of Processing on Bioactive Compounds and Nutritional Qualities of Pulses in Meeting the Sustainable Development Goal 2.” Frontiers in Sustainable Food Systems 5: 681662. 10.3389/fsufs.2021.681662.

[jfds71342-bib-0004] Choi, Y.‐M. , H. Yoon , M.‐J. Shin , et al. 2023. “Nutrient Levels, Bioactive Metabolite Contents, and Antioxidant Capacities of Faba Beans as Affected by Dehulling.” Foods 12, no. 22: 4063. 10.3390/foods12224063. 38002121 PMC10670910

[jfds71342-bib-0005] Dabhi, M. N. , V. P. Sangani , and P. J. Rathod . 2019. “Effect of Enzyme Pretreatment on Dehulling, Cooking Time and Protein Content of Pigeon Pea (Variety BDN2).” Journal of Food Science and Technology 56, no. 10: 4552–4564. 10.1007/s13197-019-03940-1. 31686687 PMC6801234

[jfds71342-bib-0006] Fernando, H. S. S. 2017. “Effects of Pretreatments on Separating the Seed Coat From the Cotyledon of Black Bean.” Master's thesis, North Dakota State University. https://hdl.handle.net/10365/28710.

[jfds71342-bib-0007] Hughes, J. , and B. Swanson . 1985. “Microstructural Changes in Maturing Seeds of the Common Bean (*Phaseolus vulgaris* L.).” Food Structure 4, no. 2: 183–189. https://digitalcommons.usu.edu/foodmicrostructure/vol4/iss2/2.

[jfds71342-bib-0008] Magrini, M.‐B. , T. Salord , and G. Cabanac . 2023. “The Unbalanced Development Among Legume Species Regarding Sustainable and Healthy Agrifood Systems in North‐America and Europe: Focus on Food Product Innovations.” Food Security 15, no. 1: 187–200. 10.1007/s12571-022-01294-9.

[jfds71342-bib-0009] Mullins, A. P. , and B. H. Arjmandi . 2021. “Health Benefits of Plant‐Based Nutrition: Focus on Beans in Cardiometabolic Diseases.” Nutrients 13, no. 2: 519. 10.3390/nu13020519. 33562498 PMC7915747

[jfds71342-bib-0010] Nguyen, T. H. , H. Tafiire , A. Van Loey , and M. E. Hendrickx . 2026. “Mechanisms of the Hard‐to‐Cook Phenomena in Common Beans: A Quantitative Microscopic Perspective.” LWT ‐ Food Science and Technology 250: 119452. 10.1016/j.lwt.2026.119452.

[jfds71342-bib-0011] Offia, O. B. , and B. U. Madubuike . 2015. “The Dehulling Efficiency and Physicochemical Properties of Pre‐Conditioned Mungbean (*Vigna radiata* (L). Wilczek) Seeds and Flour.” African Journal of Food Science and Technology 6, no. 1: 1–11. 10.14303/ajfst.2014.104.

[jfds71342-bib-0012] Olaleye, H. T. , T. O. Oresanya , and O. O. Ogundipe . 2020. “Comparative Study on Proximate and Antinutritional Factors of Dehulled and Undehulled Fermented Lyon Bean (*Mucuna cochinchinensis*).” Food Research 4, no. 5: 1611–1615. 10.26656/fr.2017.4(5).155.

[jfds71342-bib-0013] Singh, N. , P. Jain , M. Ujinwal , and S. Langyan . 2022. “Escalate Protein Plates From Legumes for Sustainable Human Nutrition.” Frontiers in Nutrition 9: 977986. 10.3389/fnut.2022.977986. 36407518 PMC9672682

[jfds71342-bib-0014] Sreerama, Y. N. , V. B. Sashikala , and V. M. Pratape . 2009. “Effect of Enzyme Pre‐Dehulling Treatments on Dehulling and Cooking Properties of Legumes.” Journal of Food Engineering 92, no. 4: 389–395. 10.1016/j.jfoodeng.2008.12.008.

[jfds71342-bib-0015] Uebersax, M. A. , K. A. Cichy , F. E. Gomez , et al. 2023. “Dry Beans (*Phaseolus vulgaris* L.) as a Vital Component of Sustainable Agriculture and Food Security—A Review.” Legume Science 5, no. 1: e155. 10.1002/leg3.155.

[jfds71342-bib-0016] Viana, L. , and M. English . 2022. “The Impact of Dehulling and Germination on the Physiochemical, Protein Solubility and Water and Oil Holding Capacities of Yellow Eye Bean (*Phaseolus vulgaris* L.) Protein Concentrates.” Frontiers in Sustainable Food Systems 6: 855788. 10.3389/fsufs.2022.855788.

[jfds71342-bib-0017] Wood, J. A. , E. J. Knights , G. M. Campbell , S. Harden , and M. Choct . 2022. “Enzyme Pre‐Milling Treatments Improved Milling Performance of Chickpeas by Targeting Mechanisms of Seed Coat and Cotyledon Adhesion With Various Effects on Dhal Quality.” Journal of the Science of Food and Agriculture 102, no. 1: 62–72. 10.1002/jsfa.11331. 34031883

[jfds71342-bib-0018] Xu, B. J. , and S. K. C. Chang . 2007. “A Comparative Study on Phenolic Profiles and Antioxidant Activities of Legumes as Affected by Extraction Solvents.” Journal of Food Science 72, no. 2: S159–S166. 10.1111/j.1750-3841.2006.00260.x. 17995858

